# Global Mapping of DNA Methylation in Mouse Promoters Reveals Epigenetic Reprogramming of Pluripotency Genes

**DOI:** 10.1371/journal.pgen.1000116

**Published:** 2008-06-27

**Authors:** Cassandra R. Farthing, Gabriella Ficz, Ray Kit Ng, Chun-Fung Chan, Simon Andrews, Wendy Dean, Myriam Hemberger, Wolf Reik

**Affiliations:** 1 Laboratory of Developmental Genetics and Imprinting, The Babraham Institute, Cambridge, United Kingdom; 2 Bioinformatics Group, The Babraham Institute, Cambridge, United Kingdom; 3 Centre for Trophoblast Research, University of Cambridge, Cambridge, United Kingdom; The Jackson Laboratory, United States of America

## Abstract

DNA methylation patterns are reprogrammed in primordial germ cells and in preimplantation embryos by demethylation and subsequent *de novo* methylation. It has been suggested that epigenetic reprogramming may be necessary for the embryonic genome to return to a pluripotent state. We have carried out a genome-wide promoter analysis of DNA methylation in mouse embryonic stem (ES) cells, embryonic germ (EG) cells, sperm, trophoblast stem (TS) cells, and primary embryonic fibroblasts (pMEFs). Global clustering analysis shows that methylation patterns of ES cells, EG cells, and sperm are surprisingly similar, suggesting that while the sperm is a highly specialized cell type, its promoter epigenome is already largely reprogrammed and resembles a pluripotent state. Comparisons between pluripotent tissues and pMEFs reveal that a number of pluripotency related genes, including *Nanog*, *Lefty1* and *Tdgf1*, as well as the nucleosome remodeller *Smarcd1*, are hypomethylated in stem cells and hypermethylated in differentiated cells. Differences in promoter methylation are associated with significant differences in transcription levels in more than 60% of genes analysed. Our comparative approach to promoter methylation thus identifies gene candidates for the regulation of pluripotency and epigenetic reprogramming. While the sperm genome is, overall, similarly methylated to that of ES and EG cells, there are some key exceptions, including *Nanog* and *Lefty1*, that are highly methylated in sperm. *Nanog* promoter methylation is erased by active and passive demethylation after fertilisation before expression commences in the morula. In ES cells the normally active *Nanog* promoter is silenced when targeted by *de novo* methylation. Our study suggests that reprogramming of promoter methylation is one of the key determinants of the epigenetic regulation of pluripotency genes. Epigenetic reprogramming in the germline prior to fertilisation and the reprogramming of key pluripotency genes in the early embryo is thus crucial for transmission of pluripotency.

## Author Summary

Large scale epigenetic reprogramming occurs in mammalian germ cells and the early embryo. The biological purpose of this reprogramming is largely unknown, although it has been suggested that it may be required for the embryonic genome to return to a pluripotent state. We carried out a genome-wide screen of promoter methylation in the mouse, comparing germ cells with pluripotent cells, multipotent cells, and more differentiated cell types. We find that promoter methylation is an epigenetic signature of developmental potency. Genes linked to pluripotency are generally hypomethylated in stem cells and hypermethylated (and silenced) in more differentiated cell types, and our genome-wide screen provides new candidates for the regulation of pluripotency. Importantly, germ cells resemble pluripotent cell types in that most promoters have been reprogrammed. However, a small group of key pluripotency regulators (including *Nanog*), are methylated in mature germ cells, presumably in order to suppress pluripotency at critical stages of germ cell differentiation. Indeed, methylation in these genes becomes reprogrammed after fertilisation so that the embryo can regain totipotency. This work, therefore, shows for the first time that epigenetic reprogramming is crucial for maintaining the pluripotency of germ and embryonic stem cells.

## Introduction

DNA methylation in CpG dinucleotides is the only known epigenetic modification of DNA in vertebrates. DNA methylation patterns are somatically heritable by virtue of the maintenance methyltransferase, Dnmt1. DNA methylation has key roles in epigenetic gene regulation and silencing, in particular in genomic imprinting, X chromosome inactivation, and silencing of retrotransposons [1]–[4]. To what extent, in general, methylation or demethylation of gene promoters plays a role in transcriptional regulation during development is still debated.

During mammalian development there are cycles of genome-wide reprogramming of DNA methylation [5]. In mouse primordial germ cells (PGCs) there is some demethylation at early stages of their development (around E8.0) concomitant with loss of H3K9 dimethylation [6]. This early reprogramming is followed by specific demethylation of differentially methylated regions (DMRs) in imprinted genes from E10.5, as PGCs enter the gonads, until E12.5, and by partial demethylation of repetitive elements [7]–[10]. A key biological purpose of demethylation during PGC development is the erasure of imprints, so that they can be replaced at later stages of gametogenesis with the appropriate gametic methylation patterns in DMRs. These reprogramming events during PGC development coincide with the re-expression of some pluripotency genes, including *Sox2* and *Nanog* [11], and the ability to derive pluripotent stem cells (EG cells) into culture. Whilst it is not known if demethylation is required for this re-expression of pluripotency genes, other genes needed for germ cell development such as *Mvh*, *Dazl*, and *Scp3* are demethylated and expressed at this time [12].

As gametogenesis progresses DNA methylation patterns are set up in a sex- and sequence-specific manner. In the male germ line this process starts prior to birth (around E15.5) for imprinted genes and repetitive elements, and is almost complete by E17.5, whilst in the female germline *de novo* methylation only commences after birth [8], [13]–[17]. Correct establishment of this DNA methylation pattern in the male germ line is vital. Abnormal hypomethylation of retrotransposons is observed in the absence of the *de novo* DNA methyltransferase *Dnmt3a* [18],[19] and of the *Dnmt3*-like gene, *Dnmt3L* [18]–[21]. Additionally, mutants in *Dnmt3a* and *Dnmt3L* have abnormal hypomethylation of paternally imprinted genes and these cells fail to progress through meiosis, resulting in infertility [20],[21]. The acquisition of *de novo* methylation pre-meiotically in the male germ line implies a need to maintain this new pattern throughout the many mitotic divisions that the spermatogonia undergo prior to meiosis.

A second major reprogramming of DNA methylation patterns occurs after fertilisation in the early embryo. Many sequences in the paternal genome such as *Line1* repeats are actively demethylated in the zygote [9],[22],[23]. Sequences in the maternal genome are passively demethylated during the cleavage divisions in the preimplantation embryo [24],[25], presumably due to the exclusion of Dnmt1 from the nucleus [26]. The purpose of methylation reprogramming in preimplantation embryos is not understood; one possible explanation is that demethylation in the early embryo is needed for the parental genomes to lose their epigenetic marks so that the embryonic genome can return to totipotency [5]. Genome-wide hypomethylation at the morula stage is then followed by lineage specific *de novo* methylation beginning at the blastocyst stage [27], presumably carried out by Dnmt3a and Dnmt3b [28]. It is possible that this *de novo* methylation leads to epigenetic silencing of key promoters during early development. Indeed some regulators of pluripotency are hypomethylated in stem cells but become methylated upon differentiation in both mouse and human [29]–[31].

In order to understand the dynamics of methylation reprogramming on a large scale we have carried out a comprehensive genome-wide analysis of promoter methylation in the mouse genome, comparing pluripotent and multipotent cell types (ES, EG, and trophoblast stem (TS) cells) with germ cells (sperm), and differentiated cells (primary embryonic fibroblasts, pMEFs). We used the recently developed meDIP (methylated DNA Immuno-Precipitation) method in combination with hybridisation to genome-wide promoter tiling arrays (NimbleGen) for this comparison [32]–[34]. Our original hypothesis was that the mature gametic genome (here exemplified by sperm) was epigenetically substantially different from pluripotent genomes (ES and EG cells). We were therefore surprised to find that the sperm promoter methylome very closely resembled that of pluripotent cells, suggesting substantial reprogramming prior to fertilisation. However, some key regulators of pluripotency such as *Nanog* were methylated in sperm, demethylation occurred after fertilisation, and this demethylation was necessary for their expression in stem cells. The overall conclusion from this work is that DNA methylation marks at key regulators of pluripotency are erased in the early embryo, a process which is critical for early development and for the establishment of a totipotent state.

## Results

### MeDIP Array Analysis of Mouse Promoters

We analysed methylation of 26,275 mouse promoters by immunoprecipitation of sheared genomic DNA with an anti-5-methylcytosine antibody and hybridisation to NimbleGen Mouse oligonucleotide promoter tiling arrays (2005-03-31_MM5). Genomic DNA was extracted from biological triplicates each of E11.5 and E12.5 EG cells, ES cells, TS cells, sperm, and primary mouse embryonic fibroblasts from E12.5 embryos (pMEFs), and was immunoprecipitated. The quality of the immunoprecipitation was determined by analysing enrichment of two genes with known methylation patterns (data not shown).

**Figure 1:**
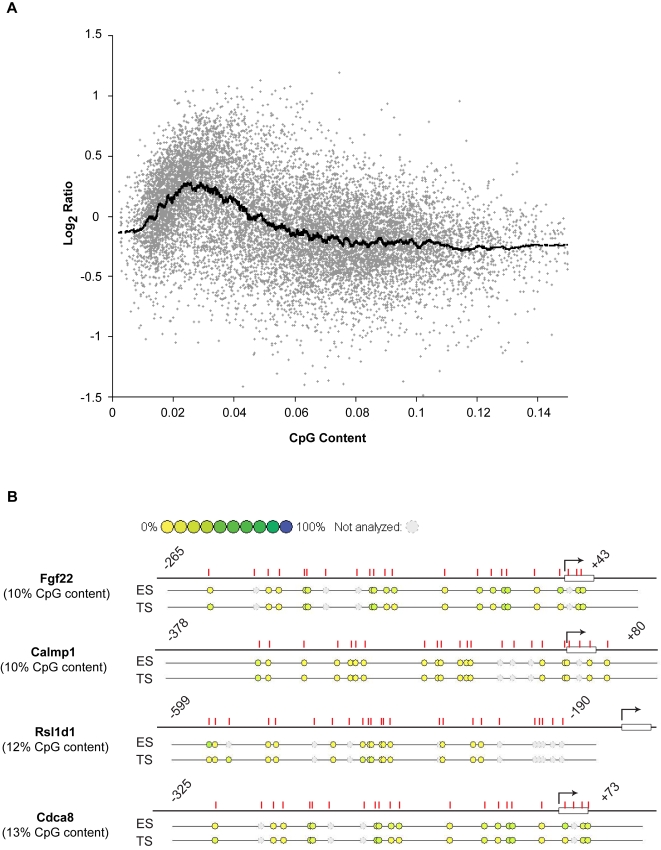
Global relationship between meDIP signal and CpG content. (A) Scatter plot of meDIP methylation signal (Log_2_ ratio) in all promoters with varying CpG content (example shown is from an ES cell sample and is representative of the pattern observed in all cell types analysed). There is an initial rise in signal up to 5% CpG content, followed by a sudden drop in signal for promoters with above 5% CpG content. (B) Promoters with more than 9% CpG content were mostly unmethylated in different cell types, as revealed by Sequenom analysis. Four examples are shown whose methylation was compared between ES and TS cells.

Examining the overall distribution of signals on the arrays we found an increase in average signal intensity between 0–5% CpG content, followed by a decrease in signal for regions between 5–9% CpG content and a flat line for regions above 9% CpG content (Figure 1A). This distribution reflects the generally hypomethylated state of CpG-rich promoters and CpG islands (above 9% CpG content), whilst we infer that relatively CpG-poor promoters can become methylated in normal tissues. The pattern observed for mouse promoters is thus similar to that recently reported for human promoters, using the same assay [34].

It has been shown that genes with very low CpG content promoters tend to be constitutively highly methylated [34],[35], while genes with high CpG content promoters often contain CpG islands which generally remain unmethylated. In agreement with this, we found that high CpG content promoters are mostly unmethylated (Figure 1B). In addition, we notice that most of the microarray signals from the high CpG content promoters failed to pass our significance test (see Experimental Procedures for details), suggesting a potential technical difficulty in measuring the methylation signals in the high CpG content regions. Therefore, we limited our subsequent analyses to a CpG content between 2 and 9%, thus excluding highly methylated CpG poor promoters (<2%) and those unmethylated promoters which contain CpG islands (>9%) (Table S1). This category of promoters corresponds to that termed ‘intermediate CpG promoter (ICP) class’ [34], in which most dynamic changes in methylation appear to occur in humans [34].

### Clustering Analysis Reveals Global Reprogramming of the Sperm Genome

**Figure 2:**
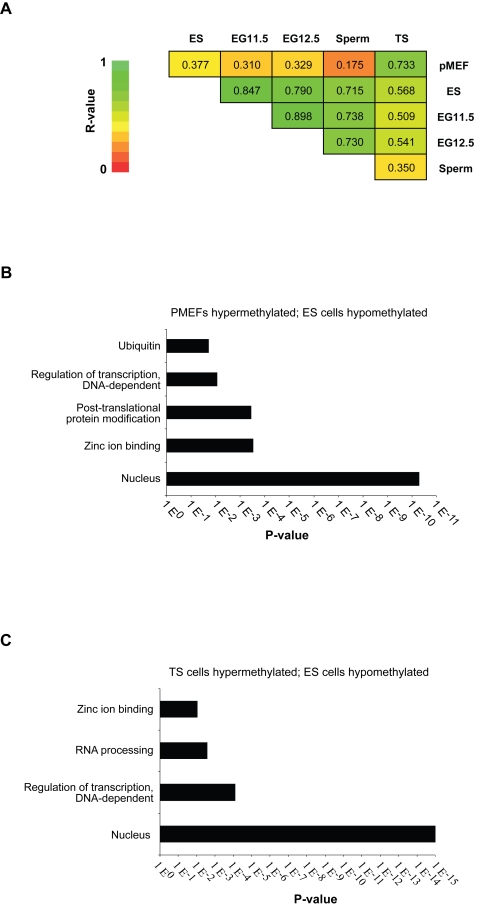
Global comparisons of promoter methylation patterns between cell types. (A) Pairwise correlation comparisons were made between all cell types to establish the similarity of promoter methylation. R-values were compared for significant correlation both within and between groups, and are represented by a colour-coded scale (green is highly correlated). (B) Gene Ontology analysis for genes which are hypermethylated in pMEFs and hypomethylated in ES cells. GO terms with a significant enrichment (p<0.01) are shown. (C) Gene Ontology analysis for genes which are hypermethylated in TS cells and hypomethylated in ES cells. GO terms with a significant enrichment (p<0.01) are shown.

**Figure 3:**
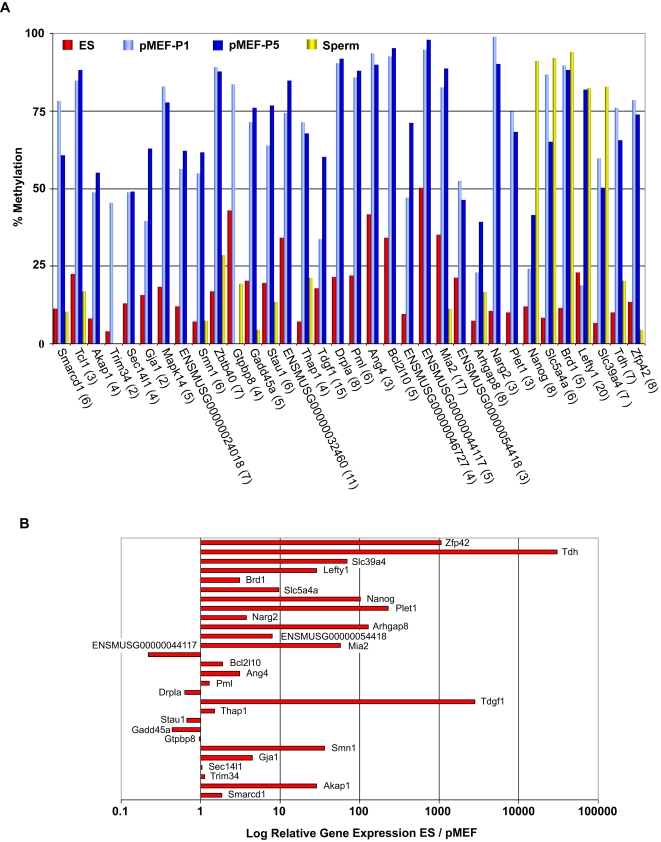
Promoter methylation and gene expression compared between ES cells and pMEFs. (A) Promoter methylation patterns in ES cells (red bars), early passage pMEFs (pMEFs-P1, light blue bars), late passage pMEFs (pMEFs-P5, dark blue bars) and sperm (yellow bars). Candidate promoter regions were identified by the meDIP screen and validated by Sequenom analysis. The number of differentially methylated CpGs analysed for each gene are given in brackets. (B) Gene expression differences between ES cells and pMEFs (P1) as determined by quantitative RT-PCR analysis. The *x*-axis gives the log-fold expression difference between the cell types (i.e., log [*ES*/*pMEF*]). Three reference genes (*Dynein*, *Rsp23* and *Hdac10-11*) were used for normalization between cell types.

ES and EG cells are pluripotent and derived from within the embryonic lineage [36]–[38], while TS cells are more restricted in their developmental potential within the extraembryonic lineage [39], and pMEFs are a committed mesodermal cell type. We were interested in comparing global methylation patterns of promoters between these cell types. To make these comparisons the ratios from the meDIP arrays were used to generate a correlation value between each pair of tissues (Figure 2A). This clearly showed that the global promoter methylation patterns of ES cells, EG cells and sperm correlate well with each other, as do those in pMEFs and TS cells. However, methylation patterns between these two groups (ES cells, EG cells and sperm versus pMEFs and TS cells) differed substantially and significantly (p<0.0001). This analysis also showed that the promoter methylation profile of TS cells is less different to that of the pluripotent cells (ES and EG cells) and sperm than the pMEF promoter methylation is, even though they are still significantly different (p<0.01 and p<0.0001, respectively). This may reflect the diverse differentiation potentials between TS cells and pMEFs. Since the potency of TS cells is restricted to the extraembryonic trophoblast lineage, it suggests that its differentiation potential is more constrained by DNA methylation than that of ES or EG cells, but somewhat less constrained than that of pMEFs. On the other hand, the tight correlation of EG with ES cells is perhaps expected given that both cell types are pluripotent, and should only differ in the methylation of the small number of imprinted genes [40] and potentially of autosomal germ line-restricted genes [35],[41],[42]. The correlation of EG and ES cells with sperm was surprising, but suggests that, as far as promoter methylation is concerned, sperm is very comparable to pluripotent cell types. Indeed, most promoters that were found hypomethylated in ES cells but hypermethylated in pMEFs were also hypomethylated in sperm, and this general pattern was confirmed by sequence-specific methylation analysis (see below, Figure 3A). We conclude that overall, promoters in sperm are already epigenetically reprogrammed and resemble those in pluripotent cell types.

This global analysis suggests that promoter methylation is an epigenetic signature of developmental potency given that the most pronounced changes occurred between ES/EG cells and pMEFs, and between ES/EG and TS cells, respectively. The global meDIP analysis provided mean signal intensity values that required correction to identify the baseline between methylated and unmethylated sequences. As an alternative we pursued a comparative approach and Gene Ontology (GO) analysis was used to identify categories within which genes differed significantly in promoter methylation patterns between ES cells and pMEFs or ES and TS cells (Figure 2B, C and Table 1). Interestingly, the majority of differences occurred in the same direction, with promoters being hypomethylated in ES cells, and hypermethylated in pMEFs or TS cells, respectively. Three GO categories (nucleus, regulation of transcription, zinc ion binding) were shared between the two comparisons between cell types, indicating that most genes that are hypomethylated in pluripotent cells are likely to act in the nucleus and be involved in transcriptional regulation. GO categories that differ between the groups may indicate molecular pathways that are more prevalent in embryonic (ubiquitin cycle, posttranslational modifications) or extraembryonic (RNA processing) lineage diversification, respectively.

**Table 1:** Gene Ontology analysis of differentially methylated genes.

GO ID	GO term	No. of Genes	Log_2_ Difference	P-value	Top 10 genes in each GO
**ES cells vs. pMEFs comparison (pMEF-ES)**					
GO:0005634	Nucleus	762	0.0827	5.15E-11	Nanog, Ihpk1, Thap1, Mrps31, Mafg, Mapk14, Grwd1, Pml, Nicn1, Tceb2
GO:0008270	Zinc ion binding	371	0.0808	2.96E-04	Thap1, Pam, Rasa2, Mpi1, Pml, Rnf2, Zfp60, Zfp113, Aebp2, Zcchc9
GO:0043687	Post-translational protein modification	289	0.0897	3.66E-04	Mapk14, Map1lc3a, Tceb2, Rnf2, Tnk1, Arih2, Npr1, Ern1, Cln3, Ece2
GO:0006355	Regulation of transcription, DNA-dependent	445	0.0647	8.89E-03	Nanog, Mrps31, Mafg, Mapk14, Pml, Tceb2, Rnf2, Zfp60, Zfp113, Aebp2
GO:0006512	Ubiquitin cycle	77	0.1545	1.93E-02	Map1lc3a, Tceb2, Rnf2, Arih2, Ube2i, Ube4a, Fbxo15, Ppil2, Usp18, Wwp2
**ES cells vs. TS cells comparison (TS-ES)**					
GO:0005634	Nucleus	763	0.0514	1.00E-15	Ihpk1, Mrps31, Thap1, Nicn1, Mafg, Zfp426, Banp, Zfp239, Rragc, Msh4
GO:0006355	Regulation of transcription, DNA-dependent	445	0.0453	8.20E-05	Mrps31, Mafg, Zfp426, Zfp239, Ankfy1, Mapk14, Nanog, Ankrd6, Snapc3, Zfp60
GO:0006396	RNA processing	68	0.0931	2.70E-03	Rragc, Ern1, Rpp30, Frg1, Papd1, Ddx56, Trit1, Sf3b1, Rg9mtd3, Nol3
GO:0008270	Zinc ion binding	371	0.0403	9.20E-03	Pam, Thap1, Kcmf1, Zfp426, Zfp239, Ankfy1, Zfp60, Rasa2, Zfp113, Zfp84

Comparisons were made between ES cells versus pMEFs and ES cells versus TS cells to establish within which GO categories the promoter methylation was most changing. The data used for the GO analysis were the subtracted average log_2_ ratios from two tissues for the 900 bp upstream of genes on autosomal chromosomes. Only promoters with a CpG content of 2–9% and which contained at least 5 probes were used. Interesting categories were judged to be those with p-value of <0.01. Where multiple nested categories were present only the most specific category (the one with the highest GO level) was kept. Genes within each category were ordered by the significance of difference in promoter methylation between the two cell types and the top 10 genes are shown as examples-these were all found to be hypomethylated in ES cells and hypermethylated in pMEFs or TS cells respectively.

### Candidate Regulators of Pluripotency Identified by Their Epigenetic Signatures

**Figure 4:**
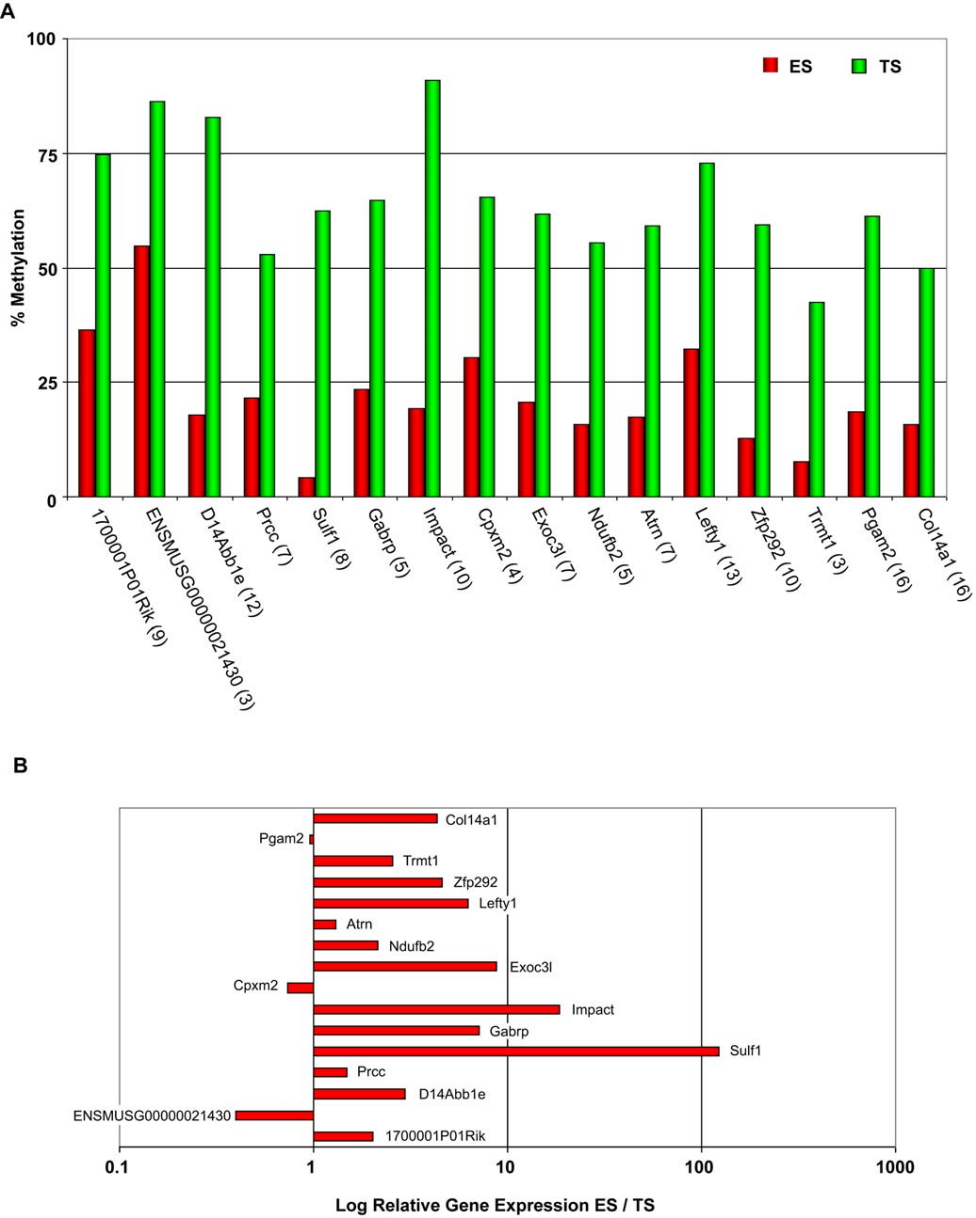
Promoter methylation and gene expression compared between ES and TS cells. (A) Promoter methylation patterns in ES cells (red bars) and TS cells (green bars). Candidate promoter regions were identified by the meDIP screen and validated by Sequenom analysis. The number of differentially methylated CpGs analysed for each gene are given in brackets. (B) Gene expression differences between ES and TS cells as determined by quantitative RT-PCR analysis. The *x*-axis gives the log-fold expression difference between the cell types (i.e., log [*ES*/*TS*]). Three reference genes (*Dynein*, *Pmm1* and *Sdha*) were used for normalization.

Having found that specific categories of genes differed in their promoter methylation between ES cells and pMEFs, and ES and TS cells, we were interested in examining individual genes in these categories in more detail. We used a more stringent algorithm than for the GO analysis in selecting potential pluripotency genes by using a sliding window approach to account for consistency of methylation signals within a number of probes in the promoter region. In addition, we applied a minimum threshold for the pMEFs and TS signal (Log2 >1.6 and >1.2, respectively) and a maximum threshold for the ES signal (Log2 <0.9) in order to select genes with the most pronounced differences in promoter methylation between ES cells versus pMEFs (Table S2) and ES cells versus TS cells (Table S3). The promoter regions of the genes predicted to be differentially methylated were subsequently validated using Sequenom MassArray methylation analysis, by which the methylated or unmethylated DNA fragments were measured quantitatively by mass spectrometry analysis. Our results showed that 88% (22/25) of candidates are validated for the ES cell versus pMEFs comparison (Figure 3A) and 100% (9/9) for the ES cell versus TS cell comparison (Figure 4A). We therefore conclude that our MeDIP experiment provides a robust predictor of differential methylation states between the cell types.

Strikingly, whilst 69 genes were predicted by our algorithm to be hypomethylated in ES cells and hypermethylated in pMEFs (Table S2), there were no genes that met the reverse criteria. *Oct4* was excluded from the analysis as it did not pass the significance filter, even though upon individual analysis some probes in its promoter were shown to be highly methylated in pMEFs. Using Sequenom MassArray technology, we were able to measure DNA methylation levels quantitatively which confirmed that these gene promoters were hypomethylated in ES cells and hypermethylated in pMEFs (Figure 3A). Similarly, examining the top ranking 70 genes from the ES cells versus TS cells comparison, we found that the great majority of genes were hypomethylated in ES cells and methylated in TS cells (67 genes) in comparison to the reverse pattern (3 genes) (Table S3). Figure 4A shows the genes from this list whose methylation patterns were validated by Sequenom MassArray. Of the genes shown in our study to be hypomethylated and expressed (see below) in ES cells and hypermethylated and repressed in pMEFs, indeed several are known pluripotency regulators and early patterning genes, including *Nanog*, *Tdgf1*, *Lefty1, Rex1 (Zfp42),* and chromatin regulators such as *Smarcd1.* The uncharacterised genes from the comparison are therefore excellent candidates for regulators of pluripotency.

**Figure 5:**
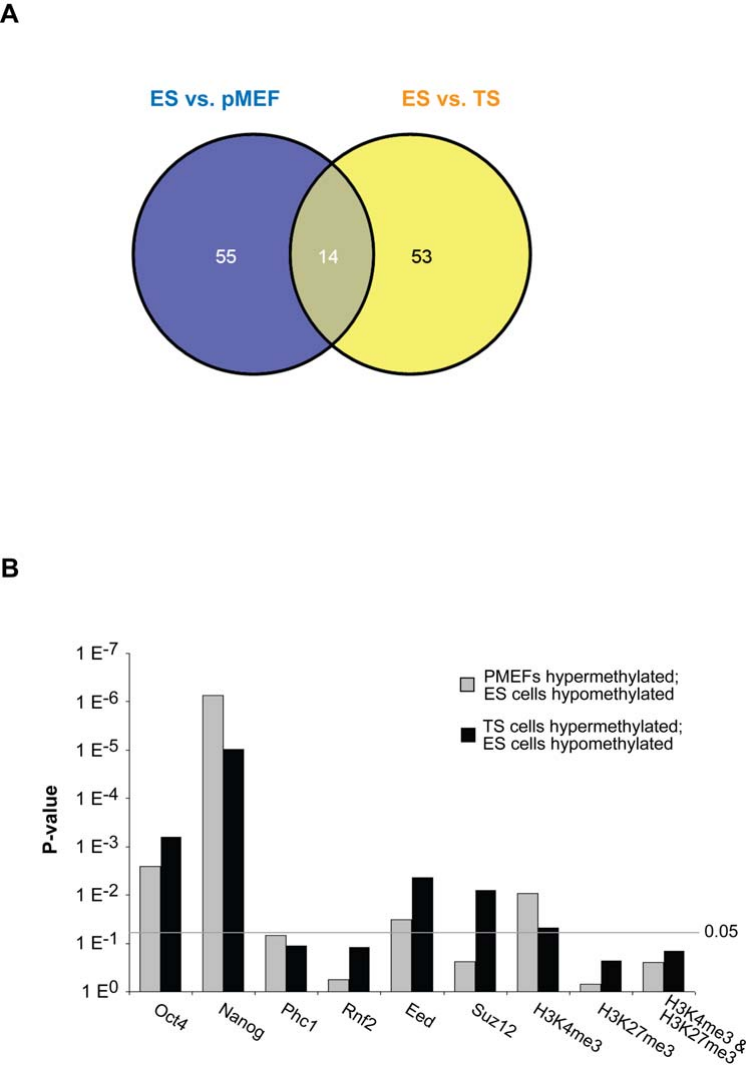
Global comparisons between promoter methylation and chromatin signatures. (A) Venn diagram showing the overlapping genes between ES cell versus pMEFs (blue) and ES cell versus TS cell (yellow) datasets. 14 genes were found in common and show hypermethylation in lineage committed and differentiated cell types. (B) Comparison of differentially methylated genes in the ES cell versus pMEFs or ES cell versus TS cell dataset with ChIP datasets of Nanog/Oct4- and PcG-binding sites [43],[45], and histone H3K4/H3K27 methylation [48] in ES cells. Correlations with p-values of <0.05 are regarded as significant. Genes analyzed were all hypomethylated in ES cells and hypermethylated in pMEFs or TS cells.

We next examined the correlation between hypermethylation of promoters and gene silencing by quantitative RT PCR of top candidates from the ES cell versus pMEFs and ES cell versus TS cell lists (Figure 3B and 4B). A substantial proportion of the differentially methylated genes showed more than 3-fold increased expression in ES cells compared with pMEFs (17 of 28 genes, 61%) or TS cells (7 of 16 genes, 44%). In contrast very few genes showed increased expression in the hypermethylated cell type (1 of 28 genes and 0 of 16 genes, respectively). Interestingly, of the approximately 70 genes predicted to differ in promoter methylation for the comparisons between ES cells and pMEFs and ES and TS cells, only 14 are common to both lists (Figure 5A, Table S4). This indicates that the differentiation pathways that are epigenetically inactivated in embryonic and extraembryonic lineages differ substantially from each other. We also analysed the developmental expression profiles of the Sequenom-validated genes on the ES cell versus pMEFs list using GNF SymAtlas. Expression data were available for 33 of the genes which are hypomethylated in ES cells and hypermethylated in pMEFs. Interestingly, the most common expression profile is one of predominant expression in either blastocysts, or in oocytes and fertilised eggs, or both (Table 2).

**Table 2:** SymAtlas expression patterns of differentially methylated genes between ES cells and pMEFs.

Gene	Oocyte and Fertilised egg		Blastocyst	
	>3	>10	>3	>10
Tcl1		•		
Akap1		•	•	
Smn1		•		
Stau1	•			
ENSMUSG00000032460		•	•	
Tdgf1				•
Bcl2l10		•		
Arhgap8			•	
Narg2		•		
Plet1				•
Nanog				•
Brd1	•		•	
Slc39a4				•
Tdh				•
Zfp42				•

Patterns of expression in blastocysts, oocytes and/or fertilised eggs of genes with differential methylation between ES cells and pMEFs (Figure 3A) as retrieved from SymAtlas [76]. Values of expression indicated are in multiples of the median expression. Where no mark is shown expression for that gene in cell type was <3 fold over the median expression. Patterns of expression relative to the median within the oocyte and fertilised egg were for these genes the same so were combined. Genes are selected from Figure 3A; only those with expression enriched in blastocysts, oocytes and/or fertilised eggs are shown in the table.

Genes that are hypomethylated in ES cells and hypermethylated in pMEFs or TS cells are potentially regulators of pluripotency. It is known that Oct4 and Nanog are key transcription factors which regulate pluripotency and self-renewal of ES cells; we therefore analysed our meDIP data for those genes found in a recent genome-wide study to be bound in ES cells by Oct4 or Nanog [43]. Significantly, genes bound by Oct4 or Nanog in ES cells become methylated in pMEFs and in TS cells (Figure 5B). Since Oct4 and Nanog are not expressed in either pMEFs or TS cells, this strong correlation suggests that DNA methylation may control the repression of the Oct4/Nanog regulatory network when pluripotency is lost.

Polycomb group (PcG) proteins are required for the maintenance of ES cell pluripotency and developmental plasticity [44]–[47]. To determine whether PcG complex occupancy is associated with DNA methylation, we compared our meDIP results to a global study of PcG-targeted genes in mouse ES cells [45] (Figure 5B). Genes occupied by key PRC1 and PRC2 proteins in ES cells were not found to be hypermethylated in pMEFs. This suggests that most of the genes targeted by PcG are silenced during embryonic development independently of DNA methylation. However, we did find a significant enrichment of genes that are hypermethylated in TS cells amongst genes occupied by PRC2 but not PRC1 complex in ES cells (Figure 5B).

To reveal any correlation between histone modifications and DNA methylation, genes with specific histone modifications in ES cells [48] were compared with our meDIP data (Figure 5B). Genes hypomethylated in ES cells (compared to pMEFs and TS cells) were found to be significantly enriched within those genes marked by trimethylated lysine 4 of histone H3 (H3K4me3). We found no significant correlations between either the repressive histone mark (H3K27me3), or the bivalent mark (H3K4me3 and H3K27me3) compared to differential DNA methylation.

### Key Pluripotency Gene Promoters Are Methylated in Sperm and Need to be Reprogrammed for Embryos to Attain Pluripotency

**Figure 6:**
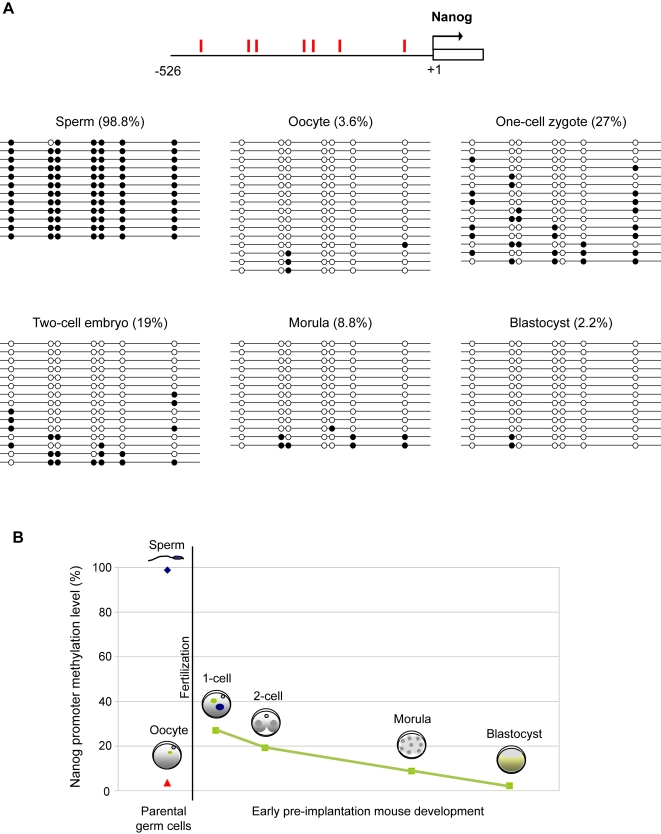
Epigenetic reprogramming of the *Nanog* promoter during preimplantation development. (A) Methylation patterns of the *Nanog* promoter in gametes and in early fertilised embryos were determined by bisulphite sequencing analysis. The *Nanog* promoter is highly methylated in sperm but hypomethylated in fertilised embryos. CpG dinucleotides are represented as open circles (unmethylated) or closed circles (methylated). The percentage of CpG methylation is indicated in brackets. (B) Summary of *Nanog* promoter methylation during preimplantation mouse development. The level of methylation at the *Nanog* promoter is given as a percentage. Methylation levels are given for the gametes and at the preimplantation stages indicating that the *Nanog* promoter undergoes both active and passive demethylation after fertilisation.

Our analysis has shown that the majority of promoters that are hypomethylated in ES and EG cells are also hypomethylated in sperm. However, there are a small number of exceptions to this rule which are interesting and important. The promoters of *Nanog*, *Lefty1*, *Brd1, Slc5a4a,* and *Slc39a4* were highly methylated in sperm while being hypomethylated in ES and EG cells (Figure 3A). In addition *Oct4* and *Sox2* are also found to be methylated in regulatory regions in sperm, albeit outside of the immediate promoter region [42]. This observation raises the question whether the promoters of these genes are reprogrammed by demethylation after fertilisation. Indeed, we confirmed by bisulphite sequencing that whilst the promoter of *Nanog* was completely methylated in sperm, it was demethylated in the zygote to an overall extent that indicates active demethylation (Figure 6A). Methylation was also analysed at later stages and the results suggest that passive demethylation occurs during further cleavage divisions until the promoter is virtually unmethylated at blastocyst stage (Figure 6A, B). This indicates that reprogramming of the *Nanog* promoter occurs by both active and passive demethylation during preimplantation development, explaining the unmethylated status of the promoter in ES cells

**Figure 7:**
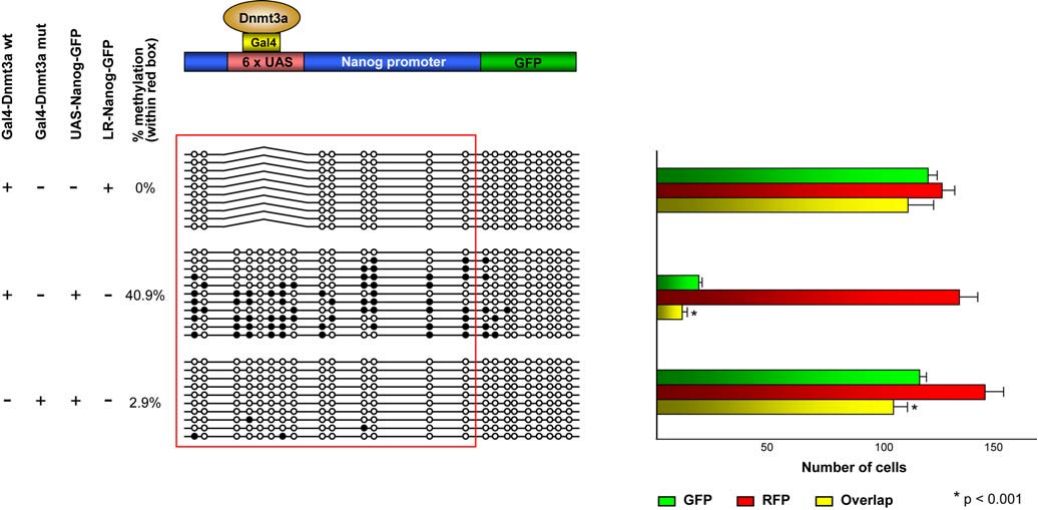
Targeted DNA methylation of the *Nanog* promoter in ES cells silences gene expression. A Nanog-GFP reporter plasmid with or without UAS targeting sequences was transfected into mouse ES cells together with a Gal4-Dnmt3a (wild-type or catalytic mutant) in addition to a pDsRed-C1 RFP construct as a transfection efficiency control. The number of GFP expressing cells (green bars), RFP expressing cells (red bars), and overlap between GFP and RFP expressing cells (yellow) was determined. Top row: transfection of Nanog-GFP without the UAS sequence together with Gal4-Dnmt3a results in high level (95.9%) GFP expression and 0% DNA methylation of the *Nanog* promoter (red box). Middle row: transfection of UAS-Nanog-GFP together with Gal4-Dnmt3a results in low level (14.5%) GFP expression and 40.9% promoter methylation. Bottom row: transfection of UAS-Nanog-GFP together with the catalytic mutant of Gal4-Dnmt3a results in high level (80.3%) GFP expression and 2.9% promoter methylation. Three independent transient transfection experiments were performed. *P* values (* indicates *p*<0.001) were calculated by Student's *t*-Test. The red box highlights the *Nanog* promoter region.

We next determined if methylation of the *Nanog* promoter inhibited its expression in pluripotent tissues. Transfection of ES cells with *Nanog-GFP* and *UAS-Nanog-GFP* promoter reporter constructs in combination with *Gal4-Dnmt3a de novo* methyltransferase expression vectors [49] was used to target methylation of the *UAS-Nanog* promoter (Figure 7). A control plasmid expressing a red fluorescent protein (pDsRed-C1 RFP) was used to normalize the efficiency between different co-transfections. Transfection of a wild-type *Nanog-GFP* reporter plasmid (LR-Nanog-GFP) with wild-type *Gal4-Dnmt3a* expression vector into mouse ES cells resulted in expression of GFP as expected (95% of RFP expressing cells expressed GFP), while co-transfection with a reporter plasmid containing *UAS* sequences (*UAS-Nanog-GFP*) led to Gal4-Dnmt3a targeted DNA methylation (41%, Figure 7) accompanied by silencing of the *UAS-Nanog-GFP* construct (14% of RFP expressing cells expressed GFP). This is in contrast to the co-transfection with a catalytic mutant of *Gal4-Dnmt3a*, where the *Nanog* promoter was neither methylated nor silenced (80% of RFP expressing cells expressed GFP). We conclude that reprogramming by demethylation is necessary for proper expression of *Nanog* in ES cells, and hence for pluripotency of the early embryo.

## Discussion

Using the meDIP technique by which methylated DNA is precipitated and hybridised onto oligonucleotide arrays [34] we have carried out the first genome-wide analysis of DNA methylation of promoters in the mouse genome in which germ cells (sperm) and pluripotent cell types (ES and EG cells) were compared with more restricted multipotent stem cells (TS cells), and more differentiated cells (pMEFs). Algorithms were developed to assess differences in promoter methylation between the different cell types, and validated using a bisulphite treated DNA and mass spectrometry based analysis (Sequenom MassArray). We find that the meDIP method combined with array hybridisation and stringent bioinformatics evaluation is a robust method for genome-wide evaluation of DNA methylation patterns.

Global comparisons between different cell types strongly suggest that promoter methylation is an epigenetic signature of developmental potency. Our study thus provides three key insights into the epigenetic regulation of pluripotency. Firstly, we find that a considerable number of gene promoters become methylated during stem cell differentiation. Secondly, our global clustering analysis shows that methylation patterns of promoters in ES cells, EG cells and sperm are surprisingly similar, suggesting that while the sperm is a highly specialized cell type, its promoter epigenome is already largely reprogrammed, resembling a pluripotent state. Thirdly, we found that while the sperm genome is overall similarly methylated to that of ES and EG cells, there are some key exceptions for which reprogramming in the early embryo is necessary.

Comparing ES cells and pMEFs, the greatest differences in methylation involve 69 gene promoters predicted to be hypomethylated in ES cells, and methylated in pMEFs. These genes are also generally predicted to be hypomethylated in EG cells by our meDIP analysis. The same holds true of the comparison between ES and TS cells; 67 genes are predicted to be hypomethylated in ES and hypermethylated in TS cells, but only 3 genes show the reverse pattern. A recent study, which compared promoter methylation in wild-type ES cells with those deficient in DNA methylation, identified a small number of genes whose promoters are methylated in ES cells [35]. Our study design, comparing different cell types, would not identify genes methylated in ES cells if they were not differentially methylated in other cell types. Hence, we find that there is a general tendency for a subset of gene promoters to become methylated in more differentiated cell types and in a lineage-specific fashion, consistent with a number of other studies which examined individual gene candidates [29]–[31]. Our study focussed on promoters with a CpG content between 2 and 9%, the so called ‘intermediate CpG promoter (ICP) class’ [34], in which most dynamic changes in methylation appear to occur at least in humans [34].

Gene expression analysis demonstrated that up to 61% of genes have promoter methylation associated with their relative gene silencing, whilst the rest of validated genes with differences in promoter methylation show little correlation in gene expression between cell types. Thus, while promoter methylation appears to be a major epigenetic determinant of gene expression, other epigenetic marking systems also have important roles to play [21],[34]. For example, a recently published promoter methylation study in ES cells suggests that demethylation is necessary but not sufficient for gene activation [35]. Furthermore, our comparisons of the meDIP data to whole genome histone modification mapping and PcG-binding studies [48] support the idea that gene repression mediated by DNA methylation or by repressive histone modifications (e.g. H3K27me3) or PcG proteins is mechanistically unconnected. The only exception to this pattern was that genes bound by polycomb complex proteins (such as Eed and Suz12) in ES cells tend to become more methylated in TS cells, suggesting that a proposed link between polycomb silencing and DNA methylation may be restricted to the extraembryonic tissues [50]. Interestingly, Nanog and Oct4 target genes were relatively hypomethylated in ES cells, and became hypermethylated during cell lineage commitment. This suggests that Nanog/Oct4 binding together with DNA hypomethylation is part of the pluripotency network to maintain an undifferentiated cell state. This class of genes was also broadly marked by H3K4me3 in ES cells, suggesting that their promoters are actively protected from *de novo* methylation [51] in pluripotent cell types, while this protection is lost during lineage commitment and differentiation.

Our screen established that transcription factors represents one of the main groups of genes being hypomethylated in ES cells, which was also observed in another recent promoter methylation study [35]. This finding is consistent with the fact that a number of transcription factors are crucial for the establishment and maintenance of the pluripotent state [52],[53], and hence our analysis may have identified additional key regulators in this network. More unexpectedly, genes belonging to the ubiquitin cycle and posttranslational protein modifications were enriched in the ES cells versus pMEFs comparison. The ubiquitin cycle is a part of the process of posttranslational protein modification and includes both deubiquitination and ubiquitination of proteins, including histones [54]. Of note is the presence of *Rnf2* (*Ring1B*), a member of the polycomb repressive complex 1 (PRC1), which mediates the monoubiquitination of histone H2A lysine 119 [55] and has recently been shown to have an important role in repressing developmental control genes in ES cells [56]. Epigenetic regulation through histone ubiquitination may be an, as yet, unexplored facet of pluripotent cell types. Differences in expression and activity of genes related to the ubiquitin cycle may also be related to a different rate of protein degradation in ES cells and pMEFs. The pluripotent nature of ES cells involves their ability to rapidly respond to stimuli such as differentiation signals. Therefore, they would be predicted to have a higher rate of protein turnover than differentiated cells, and indeed such a correlation has been found in the myogenic differentiation pathway [57].

Our meDIP data show that genes with the most pronounced methylation differences between ES cells and pMEFs have a preference for expression in early development. This suggests that early transcriptional competence is retained as hypomethylation within the cells of the ICM, and thus ES cells where expression may be reduced by other mechanisms, and subsequently permanently repressed by hypermethylation in differentiated cells. Through this comparison, we identified genes that include pluripotency factors and early patterning genes such as *Nanog* [58], *Tdgf1* [59], and *Lefty1* [60], genes involved in RNA transport with a function in germ cells such as *Akap1* [61],[62], the regulator of apoptosis, *Bcl2l10* [63], and the tumour suppressor gene *Mia2* [64]. Of particular interest are the nucleosome remodelling factor *Smarcd1* [65], and the putative bromodomain gene *Brd1* [66]. Additionally, when this comparison was evaluated against the ES versus TS cell comparison, 14 genes were found to be overlapping on the lists (Table S4). Presumably this comparison is also enriched for genes with functions in the germline, early embryogenesis, and the regulation of pluripotency. A role in these processes can therefore also be envisaged for the genes that came out of these comparisons whose function is yet to be determined.

We find that promoter methylation in sperm is strikingly similar to that in ES and EG cells. This means that the sperm genome, on the whole, has not acquired promoter methylation that would need to be erased after fertilisation to enable zygotic gene expression from the paternal genome. Thus, while the sperm itself is a highly differentiated cell type with a specialised function, its promoter methylome resembles that of other cell types of the pluripotency-germline cycle. Importantly this suggests that promoters in sperm, on a genome-wide scale, do not need to undergo extensive reprogramming by demethylation at fertilisation. This is in agreement with recently published work analysing differentially methylated regions specific to the testis [21], which tended not to be found in typical promoter regions. Gene promoters may be protected during spermatogenesis from substantial *de novo* methylation events which occur in other sequences. Large scale demethylation of the paternal genome upon fertilisation is thus likely to occur preferentially in transposons, and perhaps in inter and intragenic regions. Indeed, the Line1 family transposons (with about 100,000 members in the mouse genome) are massively demethylated upon fertilisation [9].

Whilst the global methylation of the sperm genome, as analysed by meDIP, closely resembles that of the pluripotent cell types, ES and EG cells, there are some notable and important exceptions to this rule. The promoters of genes such as *Nanog*, *Lefty1,* and *Brd1* are highly methylated in sperm; this is also observed for *Oct4*, *Fgf4*, *Fbx15,* and *Sox2* (albeit outside of the promoter) in other studies [42],[67]. These genes alone or in combination are key regulators of the pluripotent state. Hence, they may not only have to be permanently silenced in somatic cells, but also during the later stages of spermatogenesis when pluripotency is lost. This regulation might resolve a conflict within the germ line between transmitting totipotency to the next generation, whilst preventing the formation of teratomas. Reprogramming of these key genes by demethylation after fertilisation would be required to achieve pluripotency of early embryos. Indeed our experiments show that the promoter of *Nanog* is rapidly demethylated after fertilisation by both active and passive demethylation and is virtually unmethylated in the morula when its expression commences. This demethylation is necessary for expression as shown by targeting *de novo* methylation to the *Nanog* promoter in ES cells, which resulted in significant transcriptional silencing. Suppression of *Nanog* promoter activity by *in vitro* methylation has also been shown in a reporter assay in both ES and TS cells [68]. We have not examined methylation in preimplantation embryos of the other genes we found methylated in sperm; however, we suggest that they become demethylated early after fertilisation since they lack methylation in ES cells. Erasure of DNA methylation at specific key loci in the early embryo is therefore one of the epigenetic reprogramming events necessary for the establishment of the pluripotent state.

It is interesting to ask if these regulators of pluripotency are not only epigenetically silenced in somatic cells and definitive gametogenesis, but also during the allocation of PGCs in the early postimplantation embryo. For example, *Nanog* expression is silenced in early PGCs (E7.25) but is re-expressed from E7.75, coincident with the ability of deriving pluripotent EG cells into culture [11]. It is not known whether this cycle of silencing and re-expression also involves DNA methylation, but the timing of re-expression coincides with some major epigenetic erasure events in the PGCs, including DNA demethylation [6]. We therefore speculate that there might be another cycle of *de novo* methylation and demethylation in early PGCs, and that this may be required for pluripotency of EG cells.

We conclude that most promoters in the mouse genome remain unmethylated during the germline-pluripotency life-cycle. This guards the germline against acquisition of metastable epi-alleles and their transmission to future generations [69]. A small number of genes which regulate pluripotency undergo cycles of *de novo* methylation and demethylation in the germline and the early embryo, presumably to enable loss and re-establishment of pluripotency in a cyclical fashion [3]. Demethylation of the promoters of these genes is thus critical for the pluripotent part of the germline cycle, while re-methylation is crucial for the differentiation part of this cycle. Although it has been reported that a pluripotent state can be induced in differentiated cells by forced expression of a small number of key transcription factors, the efficiency of reprogramming is low and requires a long selection process [70]–[72]. Our genome-wide methylation study might thus help to identify additional factors as well as targets with a role in reprogramming and to improve the efficiency of the process.

## Materials and Methods

### Cell Lines and Other Biological Samples

ES cells (129/Sv×129/Sv-CP) F1 were cultured on a γ-irradiated pMEF feeder cell layer with ES medium (500 ml knockout DMEM, 90 ml knockout serum replacement (Hyclone), 6 ml 100x non-essential amino acids, 6 ml 100x pen/strep, 6 ml 100x glutamine, 4.6 µl β-mercaptoethanol, 1000 units/ml ESGRO (Chemicon). ES cell cultures were incubated at 37°C, 5% CO_2._ Primary mouse embryonic fibroblasts (pMEFs) were isolated from embryos at E13.5–14.5 (C57BL6/CBA) ♀ x C57Bl6♂ and cultured in fibroblast media (500 ml knockout DMEM, 50 ml foetal bovine serum (FBS), 5 ml 100x pen/strep, 5 ml 100x glutamine, 3.5 µl β-mercaptoethanol; all the reagents are from GibcoBRL, Life Technologies). Embryonic germs (EG) cells were a gift from A.Surani and were derived from PGCs from male fetuses (MF1 X Rosa) at E11.5 [73] and E12.5 [74]. TS (GFP-transgenic 129/SV and ICR) cells were grown on standard tissue culture dishes without feeder cells and gelatin coating [39] and cultured in TS complete medium with FGF4 (7 ml feeder-conditioned medium, 3 ml TS basic medium, 10 µl 1000x FGF4 stock (Sigma), 10 µl 1000x heparin stock (Sigma)) in a standard tissue culture incubator (37°C , 5% CO_2_). TS basic medium was prepared as follows: 500 ml RPMI 1640, 50 µg/ml pen/strep, 130 ml FBS, 6.5 ml 100 mM sodium pyruvate (Gibco), 1.3 ml 50mM β-mercaptoethanol, 6.5 ml 200 mM L-Glutamine. Mature sperm was collected from the caudal epididymis and vas deferens of C57BL6/CBA mice.

### Genomic DNA Extraction

Genomic DNA was isolated according to a standard protocol [75] from cultured R1 ES cells, pMEFs, sperm, TS cells, E11.5 EG cells and E12.5 EG cells. Isolated genomic DNA was purified by phenol chloroform extraction followed by ethanol precipitation, and subsequently dissolved in water overnight. The DNA concentration and quality were determined by measuring the absorbance at 260 nm and 280 nm in a spectrophotometer (Ultrospec 3100 pro, Amersham Bioscience).

### Methylated DNA Immunoprecipitation (meDIP) Assay

Genomic DNA from three biological replicates of each sample was prepared as described above. Before sonication, 20 µg of RNase were added to 60 µg of DNA in a total volume of 700 µl to digest RNA. Genomic DNA was incubated on ice and sonicated with 20% amplitude, 4 pulses with 10 s sonication and 30 s pause. 35 µl of sonicated DNA were run in 1% agarose gels to check the size of DNA fragments was in the range of 300 to 1000 bp. Sonicated DNA of the correct size was subsequently recovered by ethanol precipitation.

MeDIP was performed as described previously [33]. Briefly, 4 µg restriction enzyme digested (for subsequent PCR analysis) or 100 µg of sonicated (for genome-wide promoter array analysis) DNA was denatured for 10 min at 95°C. The denatured DNA fragments were immunoprecipitated using a monoclonal antibody against 5-methylcytidine (5meC) (Eurogentec) for 2 h at 4°C with 500 µl IP buffer (10mM sodium phosphate (pH 7.0), 140 mM NaCl, 0.05% Triton X-100). Subsequently the mixture was incubated with 30 µl of Dynabeads coated with M-280 sheep anti-mouse IgG antibody (Dynal Biotech) for 2 h at 4°C and washed three times with 700 µl of IP buffer. After recovering the pull-down methylated DNA by proteinase K digestion for 3 h at 50°C, the methylated DNA was purified by phenol-chloroform extraction followed by ethanol precipitation. The pellet was dissolved in nuclease free water (Ambion).

### NimbleGen Array Hybridisation

Genomic profiling was done by NimbleGen Systems. Arrays are composed of 1.5 kb of promoter regions for a minimal set of 26,275 mouse genes containing tiling 50-mers with 100 bp spacing (NimbleGen Systems, Inc.). Three successive early passages of R1 ES, E11.5 EG, E12.5 EG, TS cells, pMEFs, and sperm from three independent male mice older than 9 weeks were used as independent biological replicates. Six rounds of MeDIP were performed for every sample in order to obtain sufficient amounts of immunoprecipitated (methylated) DNA fragments for hybridization. We provided 3 µg of sonicated DNA as input and 4 µg of 5meC antibody pull-down DNA samples to NimbleGen Systems for differential labelling by random priming with Cy3 or Cy5 and hybridization to the mouse promoter arrays. Dye-swapping was done for one replicate of every tissue type to reduce signal error due to dye bias. Initial data preparation was performed using the in-house developed software ChIPMonk (http://www.bioinformatics.bbsrc.ac.uk/projects /chipmonk/). The raw array data were subjected to a Lowess normalisation.

### Pairwise Correlation and GO Analysis Protocol

The data used were the subtracted average log_2_ ratios from two tissues for the 900 bp upstream of genes on autosomal chromosomes. CpG content for a region is calculated as the proportion of the region +/-300bp which comprises CG dinucleotides. Only promoters with a CpG content of 2-9% and which contained at least 5 probes were used for these analyses. Firstly, for the correlation analysis, R-values were compared for significant correlation both within and between groups. Secondly, for the GO analysis, all GO categories of level ≥2 were tested. A dataset of subtracted log_2_ ratios for each gene in the category was constructed and this was tested for significant deviation from a mean of 0 using a 2-tailed t-test. T-test p-values were adjusted using Bonferoni multiple testing correction. Interesting categories were judged to be those with a corrected p-value of <0.01. Where multiple nested categories were present only the most specific category (the one with the highest GO level) was kept.

### Promoter Methylation Prediction from meDIP Data

Only promoters with a CpG content of 2–9% and which contained at least 5 probes were used, it is therefore likely that genome-representation has not been reached in this study. For the algorithm we set limits on the Log2 values to define regions we considered to be methylated and unmethylated. Regions were selected by using a 500 bp sliding window to identify areas where the methylation state consistently and significantly changed between the two tissues being compared.

### Promoter Methylation Analysis using Sequenom Technology

Genomic DNA from R1-ES, pMEF (passages 1 and 5), TS cells and sperm were bisulphite treated using the Zymo EZ DNA methylation kit (Zymo research). Candidates were selected randomly for the TS vs. ES cell comparison, and by a predominantly hierarchical approach based on the predicted methylation status in pMEFs for the pMEF vs ES cell comparison. Promoter regions were selected based on the position of the oligonucleotides on the NimbleGen promoter array and primer pairs were designed using the MethPrimer program (http://www.urogene.org/methprimer/index1.html). A complete list of primers used for analysis is available on request. Amplification of the bisulphite converted DNA, preparation of PCR products for quantitative analysis of promoter methylation detected by the Mass Array system was according to the protocol provided by the manufacturer. An example of methylation analysis using this method is shown (Figure S1).

### Quantitative RT-PCR

Total RNA was purified from 3 cell types, R1-ES, pMEFs (passages 1) and TS cells, using the RNeasy kit (QIAGEN) to eliminate contaminating genomic DNA; this was followed by DNase treatment of eluted RNA. cDNA was synthesized using SuperScript II reverse transcriptase and Oligo (dT) primers (Invitrogen) in a 20 µl reaction volume according to the manufacturer's protocol. For the PCR reactions we used Platinum SYBR Green qPCR SuperMix-UDG with ROX (Invitrogen) using the MX3005P machine (Stratagene). Reactions were done in triplicate using 1 µl of cDNA as a template in a 25 µl reaction volume. The amount of starting cDNA was normalized to three reference genes (*Dynein*, *Rsp23* and *Hdac10-11* in ES vs pMEFs; *Dynein*, *Pmm1* and *Sdha* in ES vs TS). The selected reference genes were the most consistently expressed of 12 tested references genes within the comparing cell types.

### Comparison with Other ChIP Datasets

The meDIP datasets (ES cells versus pMEFs and ES cells versus TS cells) used were under the same filtering criteria as for the GO analysis. Methylation comparisons were done using gene subsets taken from published ChIP datasets of Nanog/Oct4-binding sites [43], or PcG protein-binding sites [45], or histone H3K4/H3K27 methylation [48]. A dataset of subtracted log_2_ ratios for each gene in the ChIP dataset was constructed and this was tested for significant deviation from a mean of 0 using a 2-tailed t-test. T-test p-values were adjusted using Bonferoni multiple testing correction. A p-value of <0.01 was taken to indicate an overall shift in the methylation state in the subset of genes.

### Transient Cell Transfection

PMEF feeder cells were seeded at 5×10^5^ cells/well in six-well tissue culture plates coated with gelatin and incubated for 24 h. The growth medium was removed and R1 ES (5×10^5^) cells were plated in ES medium with LIF one day before transfection. Plasmids for co-transfection, including Gal4-Dnmt3a WT and Mut (6 µg/well), pdsRed2-C1 (1 µg/well), Nanog promoter GFP reporter plasmids including LR/Nanog-GFP, 3xUAS-NanogGFP and 6xUAS-NanogGFP (1 µg/well) were diluted with Opti-MEM I Reduced Serum Medium without serum. Transfection was carried out using Lipofectamine 2000 (Invitrogen) according to the manufacturer's protocol. Samples were analysed 2 days after transfection.

### Bisulphite PCR Amplification

Primers were designed to specifically amplify the bisulphite-converted DNA region of interest. Nested PCR was performed with PCR conditions: 94°C for 2 min followed by 10 cycles consisting of 94°C for 30 s, 50–55°C for 2 min, 72°C for 2 min, 20 cycles consisting of 94°C for 30 s, 50–55°C for 1.5 min, 72°C for 2 min plus 5 extra s for each cycle, with a final 72°C extension for 5 minutes. Primers used for nested PCR were: Nanog promoter F 5′-AATAGAGATT TTGGTAGTAAGGTTTG, R 5′-ACCCACACTCATATCAATATAATAAC; Nanog promoter nested F 5′-TTAGGGTTTGGAGGTGTAGT, R 5′-CCCACACTCATATCAATATAATAAC; Nanog-GFP F 5′-AAATAGAGATTTTGGTAGTAAGGTTT, R 5′-ACAAATAAACTTCAAAA TCAACTTA; Nanog-GFP nested F 5′-TAGAAAGAATGGAAGAGGAAATTTAG, R 5′-AATA ATAAAACAACACAATAACCAAC. Lefty1 nested PCR primers and conditions are available on request. 1–3 µl of the first PCR product was used for setting up the second nested PCR reaction.

### Microarray Database

ArrayExpress accession number E-TABM-476 provides access to our MIAME-compliant data http://www.ebi.ac.uk/microarray-as/aer/.

## Supporting Information

Figure S1 Validation of meDIP candidate, *Lefty1*, by bisulphite sequencing and Sequenom MassArray technology. The ChIPMonk profile of the promoter region of *Lefty1* in ES and TS cells is shown in the top panel. The middle line represents the median signal intensity of the array. Each vertical bar represents the methylation signal at an individual oligonucleotide probe; above the median line indicates relative hypermethylation and below indicates relative hypomethylation. Bisulphite sequencing analysis of the promoter of *Lefty1* showed that it is highly methylated in TS cells but not in ES cells, which is in agreement with the meDIP ChIPMonk pattern above. CpG dinucleotides are represented as open circles (unmethylated) or closed circles (methylated). The percentage of CpG methylation is indicated in brackets. A Sequenom profile of the promoter is shown in the middle panel. Sequenom MassArray technology gives quantitative measurements of the methylation level and is comparable to the classical bisulphite sequencing analysis. Blue circles indicate complete methylation; yellow circles indicate no methylation at individual CpG units. Examples of the MassArray spectra of a differentially methylated CpG unit upon which this colour coding is based are shown in the bottom panel. Methylation level is measured by the area ratio of methylation peak to non-methylation peak. The number of differentially methylated CpGs was counted and the average methylation level across those CpGs in each cell type plotted in Figure 3A.
(2.16 MB TIF)

Table S1 Summary of the genes analysed and the different filters which removed them.
(0.03 MB DOC)

Table S2 Gene list of ES cell versus pMEFs comparison.
(0.03 MB XLS)

Table S3 Gene list of ES cell versus TS cell comparison.
(0.03 MB XLS)

Table S4 Gene list of overlapping genes between ES cell versus pMEFs and ES cell versus TS cell comparisons.
(0.02 MB XLS)
