# Supplementary figures and images for: Global Mapping of DNA Methylation in Mouse Promoters Reveals Epigenetic Reprogramming of Pluripotency Genes
Source: PLoS Genet. 2008 Jun 27;4(6):e1000116. doi: 10.1371/journal.pgen.1000116 (PMC2432031; doi:10.1371/journal.pgen.1000116)

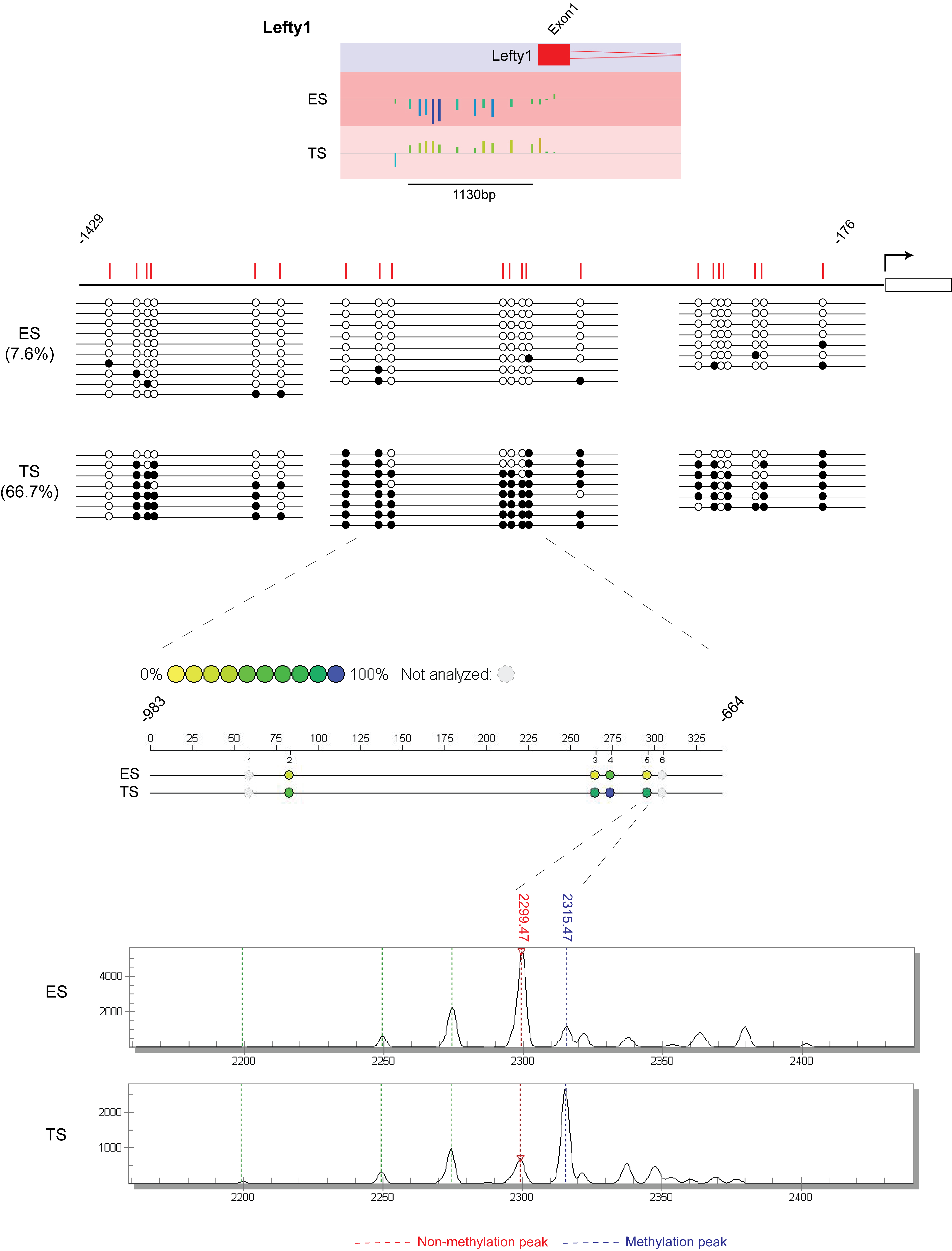

Supplement: Figure S1 — Validation of meDIP candidate, Lefty1, by bisulphite sequencing and Sequenom MassArray technology. The ChIPMonk profile of the promoter region of Lefty1 in ES and TS cells is shown in the top panel. The middle line represents the median signal intensity of the array. Each vertical bar represents the methylation signal at an individual oligonucleotide probe; above the median line indicates relative hypermethylation and below indicates relative hypomethylation. Bisulphite sequencing analysis of the promoter of Lefty1 showed that it is highly methylated in TS cells but not in ES cells, which is in agreement with the meDIP ChIPMonk pattern above. CpG dinucleotides are represented as open circles (unmethylated) or closed circles (methylated). The percentage of CpG methylation is indicated in brackets. A Sequenom profile of the promoter is shown in the middle panel. Sequenom MassArray technology gives quantitative measurements of the methylation level and is comparable to the classical bisulphite sequencing analysis. Blue circles indicate complete methylation; yellow circles indicate no methylation at individual CpG units. Examples of the MassArray spectra of a differentially methylated CpG unit upon which this colour coding is based are shown in the bottom panel. Methylation level is measured by the area ratio of methylation peak to non-methylation peak. The number of differentially methylated CpGs was counted and the average methylation level across those CpGs in each cell type plotted in Figure 3A. (2.16 MB TIF) [file pgen.1000116.s001.tif]
