# Supplementary material for: Global Mapping of DNA Methylation in Mouse Promoters Reveals Epigenetic Reprogramming of Pluripotency Genes
Source: PLoS Genet. 2008 Jun 27;4(6):e1000116. doi: 10.1371/journal.pgen.1000116 (PMC2432031; doi:10.1371/journal.pgen.1000116)
Supplement: Table S1 — Summary of the genes analysed and the different filters which removed them. (0.03 MB DOC) [file pgen.1000116.s002.doc]

Table S1 Summary of the genes analysed and the different filters which removed them.

| **Filter** | **Number of groups** | **% of starting group** |
| --- | --- | --- |
| All probe groups | 21902 | 100 |
| Groups in promoters | 13576 | 62 |
| Autosomal Groups | 12959 | 59 |
| 5 probes or more | 10174 | 46 |
| CpG content >2% | 8811 | 40 |
| CpG content <9% | 6971 | 32 |
